# Stress-inducible-stem cells: a new view on endocrine, metabolic and mental disease?

**DOI:** 10.1038/s41380-018-0244-9

**Published:** 2018-09-21

**Authors:** S R Bornstein, C Steenblock, G P Chrousos, A V Schally, F Beuschlein, G Kline, N P Krone, J Licinio, M L Wong, E Ullmann, G Ruiz-Babot, B O Boehm, A Behrens, A Brennand, A Santambrogio, I Berger, M Werdermann, R Sancho, A Linkermann, J W Lenders, G Eisenhofer, C L Andoniadou

**Affiliations:** 1 University Hospital Carl Gustav Carus, Department of Medicine III, Technische Universität Dresden, Dresden, Germany; 2 Diabetes and Nutritional Sciences, King’s College London, London, UK; 3 Center for Regenerative Therapies, Technische Universität Dresden, Dresden, Germany; 4 Paul Langerhans Institute Dresden of Helmholtz Centre Munich at University Clinic Carl Gustav Carus of TU Dresden Faculty of Medicine, Dresden, Germany; 5 Lee Kong Chian School of Medicine, Nanyang Technological University, Singapore, Singapore; 6 Center for Adolescent Medicine, UNESCO Chair on Adolescent Health Care, First Department of Pediatrics, Kapodistrian University of Athens, Aghia Sophia Children’s Hospital, Athens, Greece; 7 Divisions of Endocrinology and Hematology–Oncology, Departments of Medicine and Department of Pathology, University of Miami, Miller School of Medicine, Miami, FL USA; 8 Veterans Affairs Medical Center, Miami, FL USA; 9 Medizinische Klinik und Poliklinik IV, Klinikum der Universität München, Munich, Germany; 10 Klinik für EndokrinologieDiabetologie und Klinische Ernährung, UniversitätsSpital Zürich, Zürich, Switzerland; 11 Division of Endocrinology and Metabolism, Department of Medicine, University of Calgary, Calgary, Alta Canada; 12 Department of Oncology and Metabolism, University of Sheffield, Sheffield, UK; 13 Department of Endocrinology, Sheffield Children’s NHS Foundation Trust, Sheffield, UK; 14 Department of Psychiatry, College of Medicine, State University of New York, Upstate Medical University, Syracuse, NY USA; 15 Departments of Pharmacology and Medicine, College of Medicine, State University of New York, Upstate Medical University, Syracuse, NY USA; 16 Department for Child and Adolescent Psychiatry, Psychotherapy, and Psychosomatics, University of Leipzig, Leipzig, Germany; 17 Centre for Endocrinology, William Harvey Research Institute, Barts and the London School of Medicine and Dentistry, Queen Mary University of London, London, UK; 18 Adult Stem Cell Laboratory, The Francis Crick Institute, London, UK; 19 Centre for Craniofacial and Regenerative Biology, King’s College London, London, UK; 20 Centre for Stem Cells and Regenerative Medicine, King’s College London, London, UK; 21 Department of Internal Medicine, Radboud University Medical Centre, Nijmegen, The Netherlands

**Keywords:** Diseases, Stem cells

## Introduction

In general terms we all use the word “stress” to describe our discomfort in coping with challenges of daily life. This is mostly related to our subjective perceptions of workload and/or other unexpected physical or mental efforts we are exposed to. The term is derived from the concept of stress as a reaction to internal and external stimuli requiring acute or chronic adaptations, as introduced by Hans Selye in the second half of the last century [1–3].

In 1998 on a WHO conference on stress a more comprehensive definition of the term was provided:

“Stress may be defined as a mechanism of acute and chronic adaptation necessary for evolution and survival. The integrated stress response is part of the homoeostatic balance, and dysfunction of such response may contribute to disease. Alternations of the endocrine, neural and immune responses to stress are involved both in etiology and the pathophysiology of the most common health problems in modern society.” (World Health Organization_WHO/RPS/98.3).

In a biological sense stress is a two-edged sword representing a positive side (eustress) and a negative side (distress). On one hand, eustress helps to deal with challenges of daily life and disease, and it is also a driver of evolution and development. On the other hand, a chronic response to stress with chronic activation of the endocrine stress axis will trigger and contribute to metabolic and cardiovascular diseases [4, 5].

Endocrine and neural responses to stress have been well-defined and involve an activation of both the hypothalamic-pituitary-adrenal axis (HPA) and the sympathoadrenal system. A wide variety of external and internal stimuli, including inflammation, infection, as well as physical and mental stressors induces the release of corticotropin-releasing hormone (CRH) from the paraventricular nucleus (PVN) of the hypothalamus. CRH in turn is both a central activator of the HPA axis, as well as the sympathoadrenal system, since CRH mediates the release of adrenocorticotropic hormone (ACTH) from the pituitary and hence adrenocortical glucocorticoids as well as the release of epinephrine from the adrenal medulla [4]. In addition to CRH as a main regulator of the HPA axis there are numerous CRH and ACTH-independent factors, including neuropeptides, cytokines, the microbiota-gut-brain axis [6], and even bacterial and viral pathogens that are capable of activating the release of adrenal stress steroids [7].

Finally, central activation of the autonomic nervous system will lead to an acute activation of the adrenal medulla by the splanchnic nerves triggering the release of epinephrine and other neuropeptides. Interestingly, splanchnic nerve stimulation will also provoke the release of adrenal glucocorticoids and mineralocorticoids, which is mediated in a paracrine way by the released catecholamines [8].

Thus, there is a complex network of neuronal and cellular interactions within the end organ of the endocrine and neuroendocrine stress system. It is no coincidence that the adrenal gland combines the steroid-producing adrenal cortex and the catecholamine-producing adrenal medulla under a common organ capsule. In fact, there is an active cellular and functional interaction of cortical and chromaffin cells within the gland. Whereas adrenocortical glucocorticoids are required for the biosynthesis of adrenomedullary epinephrine, catecholamines regulate the release of steroids and the cellular function of the adrenal cortex [9]. Furthermore, patients with disorders of the adrenal cortex such as Addison’s disease or congenital adrenal hyperplasia display a dysfunction of the adrenal medulla resulting in an impaired stress response [10–12].

In addition to the cellular crosstalk between the two endocrine cell systems in the adrenal there is an important role for the vasculature and the immune system. Nearly each adrenal cell is in close proximity to endothelial cells and the gland receives ten times more blood than expected from its size [9]. Therefore, the intact physical and biochemical communication between vascular and endocrine cells is critical for the functional integrity and adaptation to stress of the entire gland, as vascular vulnerability may lead to ruptures, hemorrhage and adrenal failure with life-threatening consequences for the patient [13, 14]. Similarly, intact interactions with the systemic and resident immune cells are critical for proper functioning of the adrenal and its ability to cope with the increased stress of inflammation and sepsis [13, 14].

In addition to this complex interplay of cellular and neuronal networks in mounting and maintaining an adequate adrenal stress response, regulation of the secretion of peripheral and central stress hormones is under strict circadian and ultradian control [15]. Thus, the entire endocrine stress system is embedded in an even more complex and not fully explored cybernetic model of positive and negative feedback regulations, which mature postnatally to become fully functional only after puberty.

This leads to the obvious question of how stress in early life may shape the development and maturation of the major cellular response elements including the HPA axis. Before we try to explore this intriguing question we should reflect on what is known-up to now on the role of the classical stress hormones on the regulation of stem cells in general.

### Role of stress steroids on progenitor/stem cell populations

Progenitor and stem cell populations are both required for the successful homeostasis and adaptation of most tissues. Human hematopoietic stem and progenitor cells provide lifelong production of mature blood cells dependent on the changing requirements of each individual. Therefore, hematopoiesis is a cellular process defined by a clear balance of self-renewal and commitment to differentiation. Hematopoietic progenitors are also able to transdifferentiate into non-hematopoietic cells and exhibit overlapping genetic programs with mesenchymal and neural stem cells (NSCs). Importantly, neuronal stem cells in mice have been reported to be sensitive to steroid-induced cell death (apoptosis) through glucocorticoid receptor (GR) signaling [16], providing a model for the sensitivity of neuronal stem cells to metabolic cellular turnover and/or cellular loss induced by stress.

Interestingly, we have identified CRH1 and CRH2 receptors not only in NSCs but also in hematopoietic stem cells (HSCs) [17]. CRH receptors are involved in the systemic stress response and intriguingly CRH receptor expression is increased among immature hematopoietic progenitors but not in fully differentiated blood cells. Stimulation with CRH decreases intracellular cAMP demonstrating active signaling of this central stress hormone in HSCs [17]. Recently, the CNS has been shown to regulate embryonic HSCs via the HPA axis, as GR activation leads to HSC expansion while GR loss reduces HSC formation [18]. Likewise chronic stress exposures also activate HSC formation [19].

While, we are only beginning to understand the role of stress hormones on HSCs, their roles on NSCs and neurogenesis have been explored more comprehensively. Most importantly stress hormones exert a differential effect on neurogenesis depending on age, time, location, and nature of the exposure. Conditions that strongly elevate CRH, ACTH and glucocorticoids, such as physical activity, enriched environmental housing, or mental stress induce proliferation and survival of newborn neurons and promote neurogenesis [20]. Conversely, chronic endogenous or pharmacological exposure of NSCs to glucocorticoids has been clearly associated with reduced neurogenesis [20]. This has been linked to the activation of GRs or to changes in the expression of genes associated with cellular senescence [21–23]. Why the activation of the HPA axis leads to enhanced neurogenesis in some instances yet the loss of neural stem cells in others has not been fully understood [20].

Proliferating neuronal progenitors express higher levels of CRH receptors and are enhanced in the human fetal brain [24]. Moreover, CRH-deficient mice show reduced proliferation and increased apoptosis among neural progenitors. Thus, it has been suggested that CRH, as the major mediator of the adaptive response to stressors, could reverse damaging effects of glucocorticoids on stem/progenitor cells [24].

If CRH could reverse the damaging effects of glucocorticoids it may be assumed that the negative feedback on CRH with elevated glucocorticoid levels contributes to the reduction of neurogenesis observed during chronic stress. In addition, we and others have shown that during the acute and chronic stress of inflammation and sepsis but also in mental and metabolic disorders, several extra-hypothalamic and extra-pituitary factors including cytokines, pathogens, adipokines, growth factors, inflammatory lipids, morphogens, catecholamines, and neuropeptides can stimulate adrenal glucocorticoid release [7, 9]. Consequently, the neuroprotective effect of CRH may get lost during both acute and chronic stress. Another explanation may relate to the pattern of glucocorticoid secretion during stress as it has been demonstrated that daily oscillations in glucocorticoids control both proliferation and function of the circadian clock in the hippocampus [25]. Indeed, clock genes play a crucial role in neuronal differentiation of adult NSCs [26].

Thus, glucocorticoid regulation seems to be crucial for the maintenance of adult neurogenesis and the adaptation of NSC proliferation to environmental changes. As circadian and ultradian glucocorticoid rhythmicity is impaired during severe stress it may provide further explanation for the negative effect of glucocorticoids on neurogenesis under these conditions [15].

However, chronic activation of the endocrine stress system does not only impair neural stem cells but also other stem cell populations. For example, chronic restraint, an established model to induce chronic physiological stress in mice, leads to elevated levels of glucocorticoids and decreases the function and repair potential of mesenchymal stem cells [27]. Specifically, chronic stress inhibited their differentiation into myofibroblasts, hampering repair efficiency in a model of liver injury [27].

Considering that we are now beginning to understand the wide range of actions of stress hormones on stem and progenitor cells, the enormous clinical and therapeutic implications become obvious. Reassessing our way of thinking and our current strategy of glucocorticoid replacement regimens in those with chronic adrenal failure, we can avoid damaging effects on early cellular development, repair and regeneration by regulating cell death pathways even beyond signaling of apoptosis [28]. On the other hand we may have the opportunity to exploit the great potential of stress hormones in a precise and individualized manner for improving cell renewal and regenerative therapies.

However, before we discuss a new view on translational and clinical concepts it will be worthwhile to address how mechanistically stress induces stem cells in the endocrine organs producing the stress hormones. If stress in early life is able to direct stem cell fate and lineage commitment this can have major implications in responding to and coping with disease during adulthood. It will also add an entirely new level of complexity to the link of mental and physical stressors related to our capacity of self-renewal, cell death, resilience, cell repair, and regeneration. This in turn may have direct implications for multiple new lines of evidence regarding disease etiopathogenesis, particularly mechanisms by which early abnormalities in stress hormone regulation may lead to common diseases in later life.

### Stem cells induced by stress

An intricate network of morphogens and growth factors and a defined combinatorial code of transcription factors direct the hypothalamic, pituitary, and adrenal progenitor cells to form the mature HPA axis [29, 30]. External and internal stressors from the first day of life influence the process of cell differentiation of stem and progenitor cells in the HPA axis to form the fully functional endocrine stress system. Chronic stress in a variable stress model, which includes unpredictable stressors such as crowding, isolation, cage tilt and light-dark changes, stimulates presynaptic and postsynaptic modifications in the paraventricular nucleus of the hypothalamus that are in accordance with increased HPA axis drive [31]. Unpredictable stress activates interconnected cortical, hypothalamic and brainstem nuclei suggesting a recruited circuitry mediating chronic drive of brain stress effector systems [31].

Previously, we isolated, characterized, and differentiated chromaffin stem/progenitor cells from the human, bovine, and murine adrenal medulla [32–34]. Primary cells cultured as spheres from human medulla express progenitor markers including Nestin, NOTCH1 and SOX2 and are able to differentiate both into distinct neuron-like cell types and into endocrine chromaffin cells [32, 33]. In mice, we have identified a defined pool of glial-like Nestin-expressing progenitor cells that are multipotent and able to differentiate into both chromaffin cells and neurons both in vitro and in vivo [35]. Interestingly, immobilization stress promotes the differentiation of these Nestin-positive progenitor cells into chromaffin cells, and has also been shown to alter the expression of catecholamine-producing enzymes and the release of catecholamines [35]. Hence, stress induces both a molecular and cellular adaptation by recruiting new catecholamine-producing cells from the stem cell pool to cope with increased demand [36, 37].

More importantly, Nestin-expressing progenitors are not only located in the adrenal medulla but also in the adrenal cortex. Likewise metabolic and physical stressors seem to induce mature steroid-producing cells from this pool [38].

Tissue-specific stem/progenitor cells in the adult pituitary gland have been identified using genetic tracing experiments [39]. Cells expressing the transcription factor SOX2, a sub-population of which also express Nestin, are capable of self-renewal and the direct generation of new hormone-producing cells during postnatal life as well as in vitro [39–41]. In fact, this population can become mobilized and differentiate into the appropriate endocrine cell type in response to physiological stress [32]. In addition to their contribution to the physiological maintenance of the adult pituitary, we have demonstrated that these SOX2-expressing stem/progenitor cells can be involved in the induction of pituitary tumors [40, 41].

**Fig. 1:**
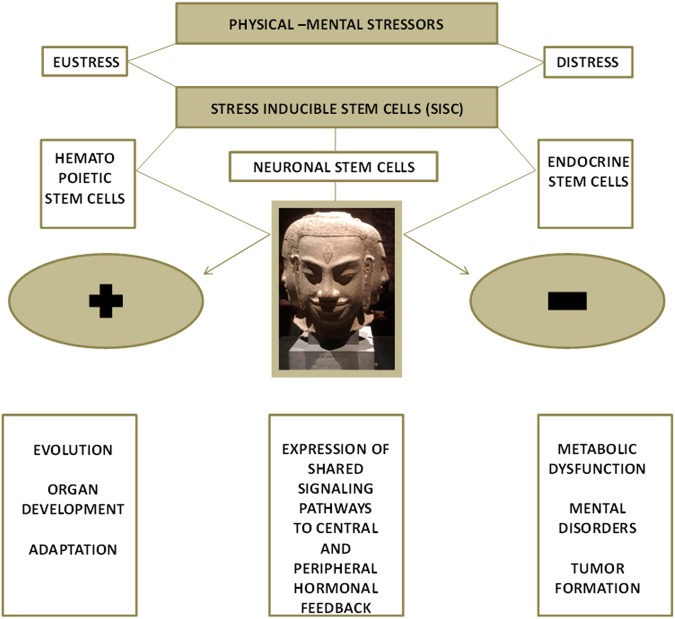
Stem cells from a range of tissues are influenced by different kinds of stress in both a positive (eustress) and negative (distress) manner giving rise to positive/negative metabolic memory

Major developmental signaling pathways are active in stem cells of the pituitary [30], and similar mechanism hold true for the peripheral effector organ of the HPA axis, the adrenal gland, in both the steroid-producing adrenal cortex and the catecholamine-producing adrenal medulla. This may suggest a uniform and coordinated signature and programming within the entire endocrine stress axis, thereby, hinting at common or shared pathways in generating stress-inducible stem cells (SISCs) shaping the entire adaptive stress response in the HPA axis and beyond. These observations also emphasize the possibility of common regulation of cellular responses within the HPA axis and sympathoadrenal system related to stress adaption. (Fig. 1)

### How is the concept of stress-inducible stem cells (SISCs) changing our view of metabolic and mental disease?

Chronic stress conditions and the inability to cope with stress lead both to increased vulnerability and aggravation of metabolic and mental disease [42]. For example, leptin and other adipocyte-derived peptides including adipocyte-derived CRH are closely linked to the endocrine stress system [43–47] and high levels of glucocorticoids produced by the adrenal cortex of subjects exposed to stress lead to reduced neurogenesis. In turn, impaired neurogenesis in the CNS is closely linked to psychiatric disease, such as depression and posttraumatic stress disorders and metabolic disorders [15, 48, 49] as angiotensin-sensitive neurons expressing CRH coordinate neuroendocrine, cardiovascular, and behavioral responses to stress [48].

Furthermore, adipocyte-derived mesenchymal stem cells from patients with metabolic syndrome are defective in differentiation, angiogenesis, motility, multipotency, as well as metabolism and immunomodulation [50]. This may suggest that a pro-inflammatory environment, with the endocrine stress axis promoting obesity through priming and dysregulation of SISCs at multiple levels (including the brain, endocrine, and adipose issues).

As already mentioned, hematopoietic stem and progenitor cells are affected by CRH and glucocorticoids [17, 18]. Therefore, the early stress activation of SISCs in hematopoiesis will induce inflammatory leukocytes contributing to the pathogenesis of metabolic-vascular disease [19].

Also in the pancreas a functional CRH receptor system is present. A brain-pancreatic islet axis is mediated by CRH and other hypothalamic peptides on beta cells [51]. Stress has also been shown to affect stem/progenitor cells in islets. This may suggest that the entire stress-regulation of stem cells in the endocrine pancreas will shape and modify lifelong islet cell mass and therefore capacity for insulin secretion [52].

Finally, glucocorticoids induce a reduction in proliferation of hypothalamic neural stem cells that can have major consequences in adulthood on the development of both mental and metabolic diseases. Hyperactivation of the HPA axis has been clearly linked to the early development of metabolic disease and to the severity of its cardiovascular complications. Glucocorticoids promote fat cell maturation, obesity, and insulin resistance while fat cell mass correlates with both glucocorticoid and aldosterone levels [53]. Also, mesenchymal stem cells isolated from metabolic syndrome and type 2 diabetes patients exhibit cellular dysfunction based on increased oxidative stress and autophagy [54]. Malnutrition, high fat diets, maternal immune activation, immobility and neglect, environmental toxins, and many other adverse events are well-known risk factors for mental and metabolic disease but also autoimmune and malignant disease will induce an early dysregulation of these intricate networks of SISCs.

This means that many longstanding observations, such as the link between early life abuse-associated trauma and subsequent metabolic risk factors/diseases [55] may now become clearer following our better understanding of the concepts of SISCs.

Twenty years after the first description of stem cells, we are able to comprehend the fact that their stress-induction represents a general concept of adaptation to the challenges of daily life and coping with disease.

Similar to the discovery and characterization of a specific stem cell pool in cancer, nature might have created a distinct subset of stem and progenitor cells that are inducible and regulated by stress. As an ancient and positive mechanism to drive evolution and development, early priming of a pool of SISCs, be it beneficial or detrimental, will later define healthy living or morbidity with premature death due to chronic and acute disease.

Stress-induction drives migration of stem cells and may explain complete or incomplete organ formation or mingling of cellular components. It may explain aberrant expression of peptide receptors with autonomous tumor formation in endocrine tissues contributing to metabolic and mental disease. In patients with both these diseases together, adrenal hyperplastic and/or adenomatous adrenals with manifest or subclinical hypercortisolism have been observed [56]. This may explain impairment of the normal circadian or ultradian rhythms of hormone release, leading to a vicious cycle of maladaptation and stress hormone exposure with all its metabolic and central consequences [15]. Such mechanisms may form a basis for the concept of a “negative metabolic memory“ contributing to irreversible long-term damage e.g., of cardiovascular disease in patients at the beginning of the metabolic disease process [57].

The observation that endocrine disruptors induce perturbations in the endoplasmic reticulum and mitochondria of human pluripotent stem cell derivatives reveals the long lasting and severe consequences of these obesogenic endocrine disrupting chemicals on the developing gut endocrine and neuroendocrine system [58]. Therefore, unavoidable environmental stressors can induce alterations in the microbiome, innate immune functions and metabolic and neural functions based on early modifications of SISCs.

### How should the concept of stress-inducible stem cells affect our clinical strategies for the diagnosis and treatment of metabolic and mental disease?

First of all it will require the development of more appropriate cellular models to study and characterize further the properties of the various subsets of SISCs. An interesting approach to analyze obesity and gene-environment interactions has been the generation of human-induced pluripotent stem cells from individuals with normal body mass index and from patients with morbid obesity (BMI >50) [59]. In this study stem cells from obese individuals were differentiated into neurons capable of secreting hypothalamic neuropeptides. This revealed functional defects in cells from morbidly obese patients including altered hormone signaling of ghrelin and leptin and dysregulated endoplasmic reticulum stress pathways [59].

Recently, we generated human-induced steroidogenic cells from fibroblasts, blood, and urine-derived cells employing forced expression of steroidogenic factor-1 and activation of the protein kinase A (PKA) and luteinizing hormone-releasing hormone (LHRH) pathways [60].

These models will allow researchers to better define the mechanisms of stress hormones and other stressors in reprogramming of progenitor cells. Furthermore, chimeric models, genome engineering and gene editing, cell encapsulation, microfluidics and organs-on-a-chip will permit the elucidation of mechanisms of stress induction of stem cells, their common pathways and relevance to chronic disease [61–69]. Regarding current treatment of metabolic and mental disease, a serious reflection on the concept of a distinct subset of SISCs in the human body will necessitate immediate changes in the management of these disorders:

1. We will have to reemphasize the enormous significance of adverse effects and stressors in early life for chronic diseases in adulthood, as childhood and adolescence represent the most dynamic periods of our development. Social chaos and family traumas can no longer be viewed as mere economic or socio-ethical problems for society; they are agents of disease causation with potentially serious and irreversible long-term health problems.
2. We will have to redesign prevention programs considering the interplay of stress, metabolic regeneration, and cell renewal.
3. The current treatment regimens of glucocorticoids have to be adapted and tailored to avoid improper triggering and dysregulation of the stress-inducible stem cell pool.
4. Current and novel medications for the treatment of metabolic and mental diseases should be tested for their effect on stress-induction of stem cells in a precise and individualized manner. Such over-arching effects on SISCs may be important for determining the relative lifetime benefits of various treatment options.
5. Regenerative therapies should be developed to exploit the benefit of eustress induction of stem cells for efficient self-renewal, repair, and recovery from chronic ailments.

## Conclusion

As the field of stem cell research is entering adulthood and as its potential for clinical applications and even for cures becomes more evident, it is the right time to embrace the new concept of stress-inducible stem cells. We propose that the effects of stress on young stem/progenitor cells during the early stages of postnatal development may predispose to adult disease. Preventing such negative effects may reduce the incidence or delay the onset of common conditions, with significant impact on welfare. Preserving the functionality of sparse stem/progenitor cell populations could have further-reaching consequences on human health than anticipated. As with other aspects of human health, the maxima of ‘prevention is better than cure’ may also apply to the stem cell field. We anticipate that more profound knowledge of the mechanism underlying the interactions between stressors and stem/progenitor cells will yield novel preventative approaches.
